# Editorial: Community series in biomarker discovery and therapeutic innovations in genito-urinary cancer management, volume II

**DOI:** 10.3389/fimmu.2026.1972929

**Published:** 2026-09-03

**Authors:** Giandomenico Roviello, Yanzhu Zhu

**Affiliations:** 1 Department of Health Sciences, University of Florence, Florence, Italy; 2 College of Animal Science and Technology, Jilin Agricultural Science and Technology University, Jilin, China

**Keywords:** biomarkers, genitourinary cancers, immunotherapy, liquid biopsy, precision oncology, targeted therapy, therapeutic innovation, tumour microenvironment

## From innovation to clinically meaningful selection

Genitourinary cancers are undergoing a rapid therapeutic transition. Immune-checkpoint inhibitors, antibody–drug conjugates, targeted agents and increasingly sophisticated molecular diagnostics have expanded treatment options, yet the central challenge has shifted from identifying active therapies to selecting the right treatment for the right patient at the right disease stage. This Research Topic was conceived to connect biomarker discovery with therapeutic innovation across prostate, bladder, renal, testicular, penile and urethral malignancies. Collectively, its contributions span circulating and tissue biomarkers, tumour–immune interactions, programmed cell death, drug development and uncommon clinical presentations. Rather than offering a single unifying marker, the Research Topic illustrates a more realistic path toward precision oncology: integration of accessible clinical measurements with genomic, transcriptomic and functional information, followed by rigorous validation in clinically defined settings.

## Accessible biomarkers and risk stratification

Several studies address the need for biomarkers that can be obtained repeatedly and implemented without complex infrastructure. In prostate cancer, Mao et al. evaluated pretreatment cytokines together with total prostate-specific antigen in 328 patients. Smoking status, total PSA and IL-8 independently associated with metastatic disease, while a parsimonious model achieved an area under the curve of 0.788. These data support the principle that inflammatory mediators may complement, rather than replace, established markers. At a different disease stage, the systematic review and meta-analysis by Liao et al. showed the prognostic relevance of baseline circulating tumour DNA in metastatic castration-resistant prostate cancer, reinforcing liquid biopsy as a quantitative measure of disease biology and burden.

Bladder cancer is particularly well represented. Two studies by Zhang et al. and Zhang et al. examined the preoperative fibrinogen-to-lymphocyte and gamma-glutamyl transferase-to-lymphocyte ratios, respectively, as inexpensive prognostic indicators in non-muscle-invasive bladder cancer. Their findings highlight the appeal of combining systemic inflammation and host status with conventional clinicopathological variables, while also underlining the need for external validation and harmonized cutoffs. Moving from peripheral blood to tumour tissue, Ding et al. assessed chromosomal instability using low-coverage whole-genome sequencing in formalin-fixed specimens from patients undergoing transurethral resection and identified a potential relationship with recurrence. Luo et al. further explored tumour biology by deriving a three-gene ribosome-biogenesis score with prognostic relevance. At the most accessible end of the spectrum, Chen et al. evaluated urinary protein semi-quantification in patients receiving PD-1-based immunochemotherapy, illustrating how a low-cost, non-invasive measurement may contribute to dynamic outcome assessment. Together, these reports define a continuum from routine laboratory indices to genome-scale measurements, and suggest that multimodal models may ultimately outperform any isolated biomarker.

The Research Topic also extends biomarker discovery to a rare malignancy. Glombik et al. investigated soluble immune-checkpoint proteins as predictors of lymph-node metastases in penile cancer. Such studies are particularly valuable where small populations make randomized evidence difficult to generate and biomarker-guided staging could meaningfully alter morbidity.

## Tumour biology, immune context and therapeutic vulnerabilities

Other contributions move beyond association to explore mechanisms that may generate new therapeutic hypotheses. In prostate cancer, Wen et al. integrated immunogenic-cell-death and ferroptosis-related genes into a prognostic model and experimentally examined TREX1, linking tumour-intrinsic stress responses to innate immunity. A subsequent study by Wen et al. investigated heterocyclic amine PhIP-associated prostate carcinogenesis using transcriptomic, machine-learning and molecular approaches, identifying candidate regulators and changes in the immune microenvironment. Complementing these original studies, Zhang et al. reviewed how compounds derived from traditional plant medicine may engage apoptosis, autophagy, ferroptosis, pyroptosis and other programmed-cell-death pathways in prostate cancer. The translational message is promising but appropriately cautious: mechanistic plausibility must be followed by pharmacologic characterization, reproducibility and prospective clinical testing.

In clear-cell renal cell carcinoma, Huang et al. demonstrated that miltirone inhibited proliferation and promoted pyroptosis through the NLRP3/AIM2/GSDMD axis in cellular models, with associated release of IL-1β and IL-18. This work illustrates the dual relevance of programmed cell death and inflammatory signalling in an immunogenic tumour. At the clinical interface, the meta-analysis by Chen et al. synthesized evidence on presurgical molecular therapy for renal cell carcinoma with venous tumour thrombus. Among 184 patients, 30.7% experienced a decrease in thrombus level and the mean thrombus-height reduction was 15.2 mm. These findings suggest potential surgical facilitation, but heterogeneity of regimens and predominantly retrospective evidence prevent assumptions about long-term oncological benefit.

Environmental and developmental dimensions are represented by Yi et al. who combined network toxicology, bulk and single-cell transcriptomics, and clinical data to explore acetyl tributyl citrate-related mechanisms in testicular seminoma. Although hypothesis-generating, this systems-level approach illustrates how exposure science may be connected with cellular heterogeneity and immune biology.

## Immunotherapy and the changing bladder-cancer landscape

Two complementary reviews place the biomarker studies within the rapidly evolving therapeutic landscape. Ning et al. discussed bladder preservation, immune-checkpoint inhibition, antibody–drug conjugates, FGFR-directed therapy and the clinical application of next-generation sequencing. Liu and Wang examined immunotherapy mechanisms, resistance, emerging targets and response biomarkers. Read together, these articles emphasize that therapeutic expansion increases, not decreases, the need for biomarker development. Biomarkers must become treatment-specific and answer concrete questions: who requires escalation, who may safely avoid toxicity, how resistance emerges, and when a molecular change should trigger a therapeutic switch.

## Rare presentations and the limits of generalization

Precision oncology must also account for anatomical and histological rarity. The report by Ma et al. described cardiac metastasis from sarcomatoid urothelial carcinoma, highlighting diagnostic complexity and the potential activity of immunotherapy in an aggressive variant. Yang et al. detailed the imaging features of primary clear-cell adenocarcinoma of the urethra and reviewed the limited literature. Case reports cannot establish efficacy or predictive biomarkers, but they preserve clinically relevant observations, alert clinicians to unusual patterns and generate questions that conventional trials may never address.

## A translational agenda

Across the Research Topic, three priorities emerge. First, discovery must be followed by independent, prospective and preferably multicentre validation, with predefined thresholds and clinically meaningful endpoints. Second, biomarkers should be evaluated within a defined treatment context; prognostic association alone does not establish predictive utility. Third, longitudinal integration is essential. Tissue genomics, circulating tumour DNA, soluble immune mediators, urine-based measurements, imaging and routine inflammatory indices interrogate different dimensions of disease and should be studied as complementary signals.

The contributions to this Research Topic capture a field moving from therapeutic innovation toward biologically informed selection. They also expose the distance that remains between promising association and routine clinical decision-making. Closing this gap will require standardized assays, transparent analytical plans, representative cohorts, functional validation and trials designed around biomarker-defined hypotheses. The next phase of genitourinary oncology should therefore be judged not only by the number of new drugs or candidate markers, but by whether they can be combined into reproducible strategies that improve survival, preserve quality of life and reduce unnecessary treatment.

